# Approaches to assessment of community level health literacy: a scoping review

**DOI:** 10.1093/heapro/daaf123

**Published:** 2025-10-22

**Authors:** Verna B McKenna, Joshua B Mathew, Yvonne Finn, Jane Sixsmith

**Affiliations:** Discipline of Health Promotion, University of Galway, Galway H91E3YV, Ireland; School of Medicine, University of Galway, Galway H91PXC2, Ireland; School of Medicine, University of Galway, Galway H91PXC2, Ireland; Discipline of Health Promotion, University of Galway, Galway H91E3YV, Ireland

**Keywords:** community level health literacy, critical health literacy, assessment

## Abstract

Addressing health literacy at the community level can provide the potential for improving health knowledge, skills, and behaviours that can ultimately lead to better health outcomes. While many tools exist for measuring individual and disease-specific health literacy, community and critical health literacy have received less attention. The review focuses on geographic communities and includes the concepts of ‘health literacy’, ‘critical health literacy’, and ‘assessment’. Key information sources for the review were Scopus, EMBASE, MEDLINE, CINAHL, ERIC, and PsycINFO, along with Google Scholar for grey literature. Hand searches of reference lists from included studies were also conducted. The search was limited to articles published between 1 January 2010, and 1 June 2023, with no restrictions on data collection methods or study design. Data were charted following guidelines from the Joanna Briggs Institute and PRISMA ScR. Initially, 14 216 citations were retrieved, with 8612 remaining after removing duplicates. Double-blind screening of titles and abstracts resulted in 33 articles, which underwent double-blind full-text screening. Three articles were included, and a hand search identified one more, totalling four articles. The small number of eligible articles and the heterogeneity of content reflect the nascent stage of development of community level health literacy and its assessment. The review findings highlight the need to develop tools to assess community level health literacy to support interventions in empowering communities in maintaining and promoting their own health.

Contribution to Health Promotion

- Much more emphasis is needed on addressing health literacy needs at the community level.
- Addressing health literacy at the community level can ultimately lead to better health outcomes, through improving health knowledge, skills, and behaviours.
- Current tools to assess community level health literacy are at a very early stage.
- Further work is needed to develop tools to assess community level health literacy.

## INTRODUCTION

Health literacy can be defined as ‘the personal knowledge and competencies that accumulate through daily activities and social interactions and across generations’ (WHO 2021). Mediated by the organizational structures and availability of resources, health literacy enables people to access, understand, appraise, and use information and services in ways that promote and maintain good health and well-being for themselves and those around them. The concept and definition of ‘community’ is central to framing this review. Community level health focuses on the collective well-being of community members who share a geographical location and who may also share health characteristics, ethnicities, and socioeconomic conditions (American Hospital Association 2025). Community level health literacy refers to health literacy-related assets (knowledge, resources, and abilities), including knowledge held by people in the community, health-promoting customs embedded in cultural beliefs and norms, and in traditional or emerging practices of daily life, and the extent to which knowledge is trusted, circulated, and adapted freely in a community (WHO 2022). The WHO recognizes community level health literacy as a key determinant of health (Nutbeam and Lloyd 2021), and a means of empowerment of people, increasing the control of communities in maintaining and promoting their own health. In addition, it advocates for health literacy development for the prevention and control of non-communicable diseases (WHO 2022). Baur *et al*. (2018) define community-based health literacy intervention as ‘any purposeful, organized activity to help a group of people find, understand, use, or communicate about health information, services, or issues for themselves or their communities’ (p. 8).

Drawing on Nutbeam’s (2000) levels of health literacy, community level health literacy is closely aligned to the critical level of health literacy, with a public health perspective and emphasis on disease prevention and health promotion in everyday life (Sørenson *et al*. 2012, Guzys *et al*. 2015). Critical health literacy can empower individuals to challenge power structures and advocate for change, while community health literacy focuses on improving the overall health literacy of a community. Abel and Benkert define critical health literacy as ‘the ability to reflect upon health determining factors and processes and to apply the results of the reflection into individual or collective actions for health in any given context’ (Abel and Benkert 2022, p. 2). Critical health literacy, therefore, facilitates improved individual resilience to social and economic adversity and, within community settings, contributes to community empowerment (Sørensen *et al*. 2012, Sykes *et al*. 2013, Sykes et al. 2018). Further, Guzys *et al*. (2015) point out that use of aggregated individual health literacy measures does not capture the ‘dynamic and often synergistic relationships within communities’ (p. 1) nor the social influences or determinants on health knowledge, beliefs, and behaviours which critical health literacy addresses, where it is an asset that can be used to gain greater control over health decision-making (Nutbeam 2008, Mårtensson and Hensing 2012) reflecting conceptualizations of community level health literacy. Addressing critical health literacy at the community level can provide the potential for improving health knowledge, skills, and behaviours that can ultimately lead to better health outcomes.

While recent years have seen a proliferation in the development of tools to measure individual, population level, and disease-specific health literacy, less attention has focussed on community level health literacy (Nutbeam *et al*. 2018a, b) or critical health literacy (Sykes *et al*. 2013). Similarly, while there are a number of tools and frameworks available to assess community health (e.g. Centre for Disease Control and Prevention 2010, American Hospital Association 2023), health literacy is not routinely addressed in these. Furthermore, a review of population health literacy assessment by Guzys *et al*. (2015) highlighted the lack of appropriate measures for assessing critical health literacy at the community or societal level.

The purpose of this study was to identify the types of assessment that are available that can provide a meaningful understanding of community level health literacy that exists in a geographically defined community setting. For this review, the authors equate critical health literacy assessment in community settings, and where the emphasis is on health literacy at the collective, rather than the individual level, to community health literacy assessment.

## RESEARCH QUESTION, AIMS, AND OBJECTIVES

The research question for this study is: what is known about the approaches used to assess community health literacy?

The aim of this scoping review is to explore approaches for the assessment of community health literacy.

Objectives:

- To identify the different approaches that have been used to assess community health literacy.
- To identify specific characteristics of the approach(es) used.
- To examine how these have been used/implemented (geographic context, community types).
- To identify barriers and facilitators in implementation.
- To examine levels of evaluation used.

## METHODOLOGY

### Overview of study design

The extent and nature of approaches available to assess community level health literacy are currently unknown. Scoping reviews are appropriate to identify knowledge gaps, scope a body of literature, clarify concepts or to explore research conduct (Munn *et al*. 2018, Tricco *et al*. 2018a, b). A scoping review was chosen as an appropriate methodology to map the available literature and to provide an overview of the evidence, concepts, and studies in the context of community level health literacy. This method adopts a rigorous methodological approach to reduce bias based on the reviewer’s viewpoint, to increase the replicability and transparency of the study, and to provide a structure for the extraction and analysis of data (Munn *et al*. 2018). This scoping review draws on methods and guidance from the Joanna Briggs Institute (JBI) methodology (Peters *et al*. 2020) and the first five stages of Arksey and O’Malley’s (2005) framework. It complies with the Preferred Reporting Items for Systematic Review and Meta-Analyses extension for Scoping Reviews Checklist (Tricco *et al*. 2018a). This details 20 essential reporting items in order to synthesize data and report evidence in a systematic and coherent manner.

### Ethical approval

Ethical approval was not required for this scoping review, which focused on mapping the literature rather than primary data collection.

#### Eligibility criteria

The Population, Concept, Context (PCC) framework is recommended as a guide to construct clear and meaningful objectives and eligibility criteria for a scoping review (Pollock *et al*. 2023). In addition, it was used to guide the specific study characterizes for inclusion in this scoping review as recommended by Munn *et al*. (2018). The population of interest in this study is communities from a geographic perspective (meaning that it is defined by its geographic boundaries, e.g. a city, town, and neighbourhoods).

The concepts include ‘community health literacy’, ‘community level’, and ‘assessment’ (see Table 1 for all concepts used in search strategy). As only studies published in English are eligible, this limits the geographical context to those in English speaking regions. An iterative approach was used to allow for the development of inclusion and exclusion criteria in the context of the presenting evidence (Booth 2008, Gough *et al*. 2012).

**Table 1.** General search strategy and term combinations.

**Concept 1:** ‘Critical health literacy’ OR ‘Public health literacy’ OR ‘Community health literacy’	**AND**	**Concept 2:** Communit* (-y, -ies) OR ‘Community-based’ OR ‘Community level’ OR ‘Community setting’	**AND**	**Concept 3:** ‘Assessment’ OR Measure* (-ment, -ing) OR Framework OR Evaluation OR Approaches OR Tools OR Diagnosis OR Profil* (-e, ing) OR Audit OR Method* (-ology) OR Outcomes

#### Inclusion criteria

All primary and secondary research was included that met the following criteria: articles describing how community level health literacy was measured/assessed. No limit was placed on the methods used for data collection or on study design used. The grey literature was also included. Searches were limited to articles published between 2010 and 2023 as this period corresponds with an increase in research on the concept of health literacy and emergence of a focus on health literacy at the community level (Nutbeam Levin-Zamir and Rowlands. 2018a, Nutbeam McGill and Premkumar 2018b).

#### Exclusion criteria

Articles examining the assessment of individual level health literacy were excluded. Articles published before 1 January 2010 and after 1 June 2023, were excluded. Articles published in languages other than English were also excluded.

### Information sources, search, and selection of sources of evidence

A search strategy that incorporated filters and the Medical Subject Headings, and key-text terms was designed by V.B.M., Y.F., and J.S. (see Table 1).

Electronic databases accessible through the James Hardiman Online Library, University of Galway were identified and used to search for relevant literature. Database selection involved an iterative process, including familiarization with other reviews in the field of health literacy and community (e.g.: Baur *et al*. 2018, Platter *et al*. 2019, Sawyers *et al*. 2022), discussions among authors and discussions with a university librarian to finalize the search strategy The initial search strategy was then piloted in the Medline database and yielded 13 258. Following familiarization with the pilot search results by three of the authors (V.B.M., J.S., and Y.F.), it was agreed to remove the search terms of ‘health literacy’ and ‘community health education’ from Concept 1, leaving the remaining three search terms as per Table 1. No changes were made to Concept 2 or Concept 3 (Table 1). Nine databases were searched, namely, Scopus, EMBASE, MEDLINE, CINAHL, ERIC, PsycINFO, ProQuest (Dissertations and Theses Global; Public Health), The Cochrane Registry and Web of Science. The grey literature was also searched using the following sources: Goole scholar, Lenus (The Irish Health Repository), Open Aire, Irish government resources (e.g. Health Service Executive; Department of Health), World Health Organization (WHO) and US Centres for Disease Control and Prevention.

The search strategies and search yields were piloted to ensure the retrieval of relevant titles. The databases were searched using the finalized key terms in combination with Boolean operators as set out in Table 1. The search strategy was adapted for each database and its implementation in the Medline database is provided in Supplementary Appendix 1.

**Figure 1.**
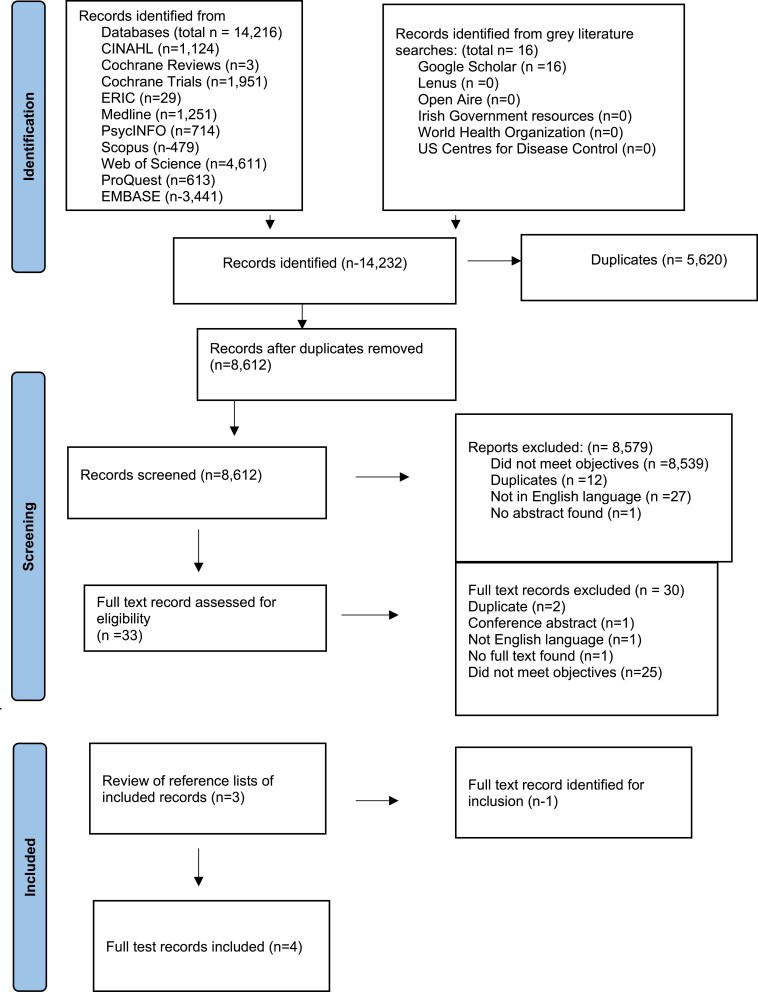
PRISMA flowchart.

Following each database search, the results were saved and uploaded to Rayyan (Ouzzani et al., 2016), a free online tool that is used to support title and abstract screening for systematic reviews (Harrison *et al*. 2020). This tool assisted in the blind review process for multiple screeners. A two-stage screening process was employed to determine the relevance of the articles. Initially the title and abstract were screened with irrelevant articles and duplicates removed. All titles and abstracts were double blind, screened by J.M./V.B.M. and J.M./Y.F. Where consensus was not reached on the eligibility status of an article, it was discussed by all of the authors and consensus on inclusion or exclusion reached. Reasons for exclusion of articles were recorded (see Fig. 1). Studies where any aspect of the PCC matched the key concepts of the research question were added to the eligible list for further review.

The second stage of screening involved reading each article in full to determine its fit with the PCC eligibility criteria. This stage also involved a double-blind review as outlined for stage one. The reference lists of eligible studies were then screened to identify any additional relevant studies. The full screening and selection process are displayed in the PRISMA flow chart (see Fig. 1).

### Review process and data charting

The data extraction and charting phase involves synthesizing, interpreting, and sorting material into key themes and issues (Arksey and O’Malley 2005). The extraction form was collated based on the JBI template for sources of evidence details, characteristics, and results extraction instrument (Peters *et al*. 2020), with reference to Pollock *et al*. (2021) practical guide to scoping reviews. The data charting form was developed drawing on categories, as agreed by the research team. Data was abstracted on article details (title, year of publication, origin country, authors), aim/purpose of the research, population and sample size, methodology with methods (e.g. focus group, interviews), intervention type, outcomes, and key findings relating to the scoping review purpose. An Excel spreadsheet was used to chart the data (see Table 3).

### Critical appraisal of individual sources of evidence

Narrative critical appraisal drawing on guidance from the JBI Manual for Evidence Synthesis (Peters *et al*. 2020) using the ‘text and opinion’ checklist, developed by McArthur *et al*. (2015), was applied to the studies identified. This checklist includes reference to the analytical process and logic of the opinions set out.

The aim of the critical appraisal process was to assess the risk of bias in the identified articles. A double-blind approach to this quality assessment was undertaken by Y.F. and J.S. with each assessing the identified studies independently before comparing results. The process including planning for any discrepancies to be discussed and mediated by a third author (V.B.M.) should this be necessary for consensus to be reached.

### Data synthesis

Data is reported for each selected study within each category as identified in the data extraction process. Findings are described in Table 3. References were synthesized during searches using a reference manager tool, Zotero (Corporation for Digital Scholarship 2023) and Rayyan Ouzzani, Hammady, Fedorowicz, and Elmagarmid (2016). Charted data were synthesized using narrative methods and descriptive statistics using Microsoft Word and Excel.

## RESULTS

### Selection of sources of evidence

The initial implementation of the search strategy across databases and other data sources retrieved 14 216 citations; of which 5620 duplicates were excluded, resulting in 8612 records. Following review of the title and abstract, 33 articles were found to be eligible for full-text review. Following the full-text review, three of the 33 articles were selected for inclusion. The remaining 30 articles were excluded as they did not meet the scoping review objectives. Please see Fig. 1 for complete details on reasons for exclusion. A hand search of the references of three full-text articles was completed and identified one further record to be included. Therefore, the final number of included records was four. A large proportion of records excluded through the review process (after duplicates were removed) were due to records not meeting the scoping reviews objectives, with many identified records examining the assessment of individual level health literacy.

The search strategy process and results of its implementation are presented in Fig. 1 with reference to reasons for exclusion of records during title and abstract review and during full-text review.

### Characteristics of source of evidence

The characteristics of the four articles found to be eligible for inclusion in this scoping review are described below with some information tabulated in Table 2 drawing on the JBI template for scoping reviews by Pollock *et al*. (2023).

**Table 2.** Characteristics of source of evidence.

Paper	1	2	3	4
Author(s)	Platter, H., Kaplow, K., and Baur, C.	Platter, H., Kaplow, K., and Baur, C.	Guzys, D., Kenny, A., Dickson-Swift, V. *et al*.	Im, H., and Swan, L. E.
Title of source	The Value of Community Health Literacy Assessments: Health Literacy in Maryland	Community Health Literacy Assessment: A Systematic Framework to Assess Activities, Gaps, Assets, and Opportunities for Health Literacy Improvement	A critical review of population health literacy assessment	Qualitative exploration of critical health literacy among Afghan and Congolese refugees resettled in the USA
Publication	Public Health Reports	Health literacy research and practice	BMC Public Health	Health Education Journal
Year	2022	2019	2015	2019
Date data collected	Mid-February to early April 2018	Mid-February to early April 2018	Not reported	Not reported
Type of evidence source	Primary research: peer-reviewed research article	Primary research: peer-reviewed research article	Evidence syntheses: peer-reviewed narrative review	Primary research: peer-reviewed research article

Of the four identified and eligible articles, one study was a critical narrative review of population health literacy assessment (Guzys *et al*. 2015). The second study, study by Im and Swan (2019) reported a component of a community-based participatory research project comprising a qualitative exploration of critical health literacy. The remaining two articles are linked, with the same authors reporting on a follow-up on the original work. The first reports the process of implementing a community level health literacy assessment developed based on established community health assessment principles. This process undertook an environmental scan and qualitative interviews with members of community organizations followed by a forum of participants and public health professionals where initial findings were disseminated and agreement on the results assessed (Platter *et al*. 2019). The second reports the community level health literacy assessment results in detail using the developed community health literacy assessment framework (Platter *et al*. 2022).

The population and context of the study by Im and Swan (2019) comprised refugee organizations and members of the Afghan and Congolese resettled refugee communities in Virginia in the USA. The studies by Platter *et al*. (2019, 2022) were undertaken with the communities, through community organizations with public health professionals, and in the context of 24 counties in Maryland, USA.

The concepts in each of the studies were health literacy, critical health literacy, and assessment (Guzys *et al*. 2015, Im and Swan 2019, Platter *et al*. 2019, 2022).

### Critical appraisal within sources of evidence

A blind independent review of the four included articles by two reviewers (Y.F. and J.S.) concurred for all aspects in each article, discussion and mediation were not necessary.

### Results of individual sources of evidence

Table 3 presents information on the results of the individual sources of evidence including outcomes and key findings.

**Table 3.** Charting of data with results.

Title Year of publication Origin/country of origin	Author(s)	Aims/purpose	Population and sample size	Methodology/methods	Intervention type, comparator and details of these, e.g. duration of the intervention) (if applicable)	Outcomes and details of these (e.g. how were they measured)	Key findings that relate to the scoping review question/s
The Value of Community Health Literacy Assessments: Health Literacy in Maryland 2022 USA	Platter H, Kaplow K, Baur C.	To describe county and regional health literacy activities, assets, gaps and opportunities in Maryland.	57 participants across 56 organizations representing 24 counties in Maryland	Environmental scan (using developed Community Health Literacy Assessment to identify community resources, health indicators, and organizations Qualitative interviews with participants from local health improvement coalitions and health departments. Content analysis used to review, quantify and interpret interview data	N/A	Data collected on multiple dimensions of health literacy with three main themes identified: health literacy assets and activities, health literacy gaps, and health literacy opportunities. The most cited asset was collecting data to inform health literacy practices, the most cited gap was using jargon with community members, and the most cited opportunity was increasing public awareness of existing health programmes through improved outreach and teaching health information–seeking behaviours	This study demonstrates that a systematic community health literacy assessment is a feasible way to collect large amounts of health literacy data. The community health literacy framework can be used to operationalize health literacy as a health indicator and include as a community health assessment measure
Community health literacy assessment: a systematic framework to assess activities, gaps, assets, and opportunities for health literacy improvement 2019 USA	Platter H, Kaplow K, Baur C.	To collect information about county and state level health literacy gaps, assets and opportunities. To characterise the status of health literacy in Maryland. To establish an initial baseline for county and state strategic planning and future collaboration. To learn how county level organizations rank themselves on their use of health literacy practices. To create a network of public health professionals dedicated to sharing and improving health literacy practices and examples across Maryland	Interviews with 56 individuals from 49 organization. 40 public health professionals (including 10 interview participants) attended the forum	Community health literacy assessment implemented form Jan 2018-April 2018. Environmental scan of each county in Maryland identified health indicators, community resources, and health organizations or professionals. Representatives of organization participated in interviews about their health literacy work. Interviews analysed to identify themes and to summarize and quality perspectives by county. A forum with interview participants and public health professionals was convened to assess the findings and disseminate initial results	N/A	Themes of health literacy definitions and organizational ranking on the use of health literacy best practice	The need to be flexible with the interview approach, importance of performing member checking and allowing participants to self-define health literacy. Findings indicate that a small team can perform a large scale –assessment that provides actionable information at state and county levels. Results can influence future interventions, inform strategic planning and collaboration, and lead towards developing a health literate society
Qualitative exploration of critical health literacy among Afghan and Congolese refugees resettled in the USA 2019 USA	Im, H. and Swan, LET	To identify critical HL skills and competencies that are practiced in refugee communities and explore impact of community health workshops	25 Afghan and Congolese refugees	Community-based participatory research. Focus group (FG) interviews were integrated into community health workshops	Weekly community health workshops over 2 months; these were delivered separately to each group i.e. with Afghan group first and then with Congolese group No comparator	Critical health literacy skills and competencies Hybrid thematic analysis of FG interviews	Critical health literacy domains in (refugee) communities identified were: Critical appraisal Self-efficacy and confidence Empowerment Problem- solving and collective action
A critical review of population health literacy assessment 2015 Australia	Guzys, D., Kenny, A., Dickson-Swift, V., and Threlkeld, G	To examine commonly used assessment tools to determine their appropriateness for assessing the critical health literacy of population groups	N/A	Critical Review	N/A	Commonly used health literacy assessment tools identified and critiqued in the context of use in community settings and with population groups	A public health approach, founded on health promotion theories with public involvement provides a useful scaffold to assess the critical health literacy of population groups

### Synthesis of results

#### Introduction

The identification and eligibility for inclusion of four articles limits synthesis of results; nevertheless their content is described in relation to the scoping reviews aim and objectives. Conceptually, population health literacy assessment is examined through a critical review of previous developments to inform advancement in the area by Guzys *et al*. (2015) In the article by Im and Swan (2019) refugee community members are enabled to identify four major thematic themes of community level health literacy which, they state, could be used for future research and assessment. Platter *et al*. (2019, 2022), in their two concomitant studies, develop a community level health literacy assessment framework which links with established community health assessment processes which on implementation was found to be feasible.

#### Approaches to assessment

The identification of approaches to community level health literacy assessment is possible only tentatively due to the limited articles and the stage of development of the area, nevertheless, an indication of an approach proposed or taken in the area at this stage is discernible. Guzys *et al*. (2015) identifies that work in health literacy to date draws on education, communication, and healthcare experts and recognizing gaps in these approaches proposes the use of a public health approach based on health promotion as a scaffold for future developments. Platter *et al*. (2019, 2022) reference public health and draw on established and implemented community health needs assessment tools and Im and Swan (2019) reference a public health approach using community engagement and participation.

The articles take different approaches to the development of assessment. Guzys *et al*. (2015) through critical review from a public health perspective, examines health literacy assessment tools in the context of the evolving definitions of health literacy with the objective of contributing to development in the field, specifically in assessment of critical health literacy of population groups. Im and Swan (2019) in the context of addressing healthcare challenges, take a bottom-up approach, collaborating with refugee communities to identify and explore community health literacy skills and competencies practiced by community members to inform developments. The articles by Platter *et al*. (2019, 2022) present research which responds to the US National Action Plan to Improve Health Literacy (US Department of Health and Human Services 2010), and as such takes a top-down approach, identifying and collating information already gathered and available through an environmental scan and document review. Interviews with individual representatives of community organizations were also undertaken with discussion of results through a forum with participants and public health professionals.

#### Characteristics of the approaches

Several characteristics of the four articles in relation to development of community level health literacy assessment are discernible. There is identification of a focus on individual and functional health literacy (Guzys *et al*. 2015, Im and Swan 2019), with recognition of the use of an aggregate of individual health literacy assessments used for population groups (Guzys *et al*. 2015, Platter *et al*. 2019) which is limited. This contributes to the identified need for further development for community level health literacy assessment in each article (Guzys *et al*. 2015, Im and Swan 2019, Platter *et al*. 2019, 2022).

The inclusion of community members in developments of community health literacy assessment is referenced in these articles. In the study by Im and Swan (2019) with the research conducted as part of an established university-community partnership, community members were engaged as active participants and were integral to the research process. In the conclusion of the review by Guzys *et al*. (2015) the need for inclusion of community members throughout the research process in the development of a framework to assess critical health literacy in population groups is identified. Platter *et al*. (2019) recognize that an ideal process of assessment would include community members as full participants in all aspects of the process. Although it does not appear that community members were participants in the development or implementation of the community health literacy assessment framework (Platter *et al*. 2019, 2022).

#### Implementation of assessments

In relation to implementation, Platter *et al*. (2019, 2022) incorporated health literacy into established community health assessment activities and used public health and healthcare data already gathered for community health purposes to contribute to the development of the community level health literacy assessment. This information was supported by interviews with members of community organizations representing the populations of 24 counties in Maryland, USA. Similarly, Im and Swan (2019) built on an established university—community partnership to explore community health literacy with members of the resettled Afghan and Congolese communities, a vulnerable community group, in Virginia, USA.

#### Implementation barriers and evaluation

The developmental stage of the research means that barriers to implementation of community level health literacy assessment were not explored. The research area is not developed enough to consider evaluation overall. However, the article by Platter *et al*. (2022) does report that the community health literacy assessment devised was found to be feasible.

## DISCUSSION

Four articles published in peer-reviewed journals were found to be eligible and were included in this scoping review. This indicates a dearth of studies in the area of community level health literacy assessment which compromises consideration of the results. Nevertheless, with that caveat, what is described in these four papers will be considered in relation to the purpose of this scoping review. Initially a summary of the articles is provided followed by consideration of their content in relation to the aim and objectives posed in this scoping review.

Guyzs *et al.* (2015) provides consideration of population health literacy assessment through a critical review of the literature in the area. They conclude that the focus of health literacy assessment should move to a focus on critical health literacy at a societal level and that Health Promotion can provide a useful scaffold for the development of an assessment framework. Im and Swan’s (2019) primary research, through engaging with the community, explores critical health literacy with a resettled refugee population as part of a community-based participatory research project. Community health literacy thematic domains identified by participants were critical appraisal, self-efficacy and confidence, empowerment and collective problem solving. Platter *et al*. (2019) provides information on the development of, and presents the process of implementation of, the Community Health Literacy Assessment framework, to assess gaps, assets, and opportunities to improve health literacy practices. Results include identification and ranking of health literacy best practices (use of educational videos, educational materials, in-person community outreach, raising provider awareness for health literacy; provider health literacy training, interpreter services, multiple languages and community input and engagement) and identification of health literacy definitions used by organizations. Their follow-on article (Platter *et al*. 2022) undertakes further analysis of data collected in the first study and concludes that a systematic community health literacy assessment is a feasible way to collect information on community health literacy. In addition, the authors report that the most cited health literacy opportunity involved increasing public awareness of existing health programmes. Other geographic locations could feasibly replicate the framework to assess community level health literacy.

Overall, the primary research from the USA reported in three of these four articles is situated in the development stage of community level health literacy assessment and initial implementation of a community health literacy assessment framework. This contrasts with developments and research undertaken in the context of medicine and healthcare on individual functional health literacy assessment (Altin *et al*. 2014). This discrepancy (between research activity in individual functional health literacy and critical health literacy in communities) has been identified previously (Guzys *et al*. 2015). Our scoping review findings agree with this observation, where many studies measure individual health literacy of community dwellers as a proxy for measurement of critical health literacy and/or community health literacy. This is a methodological weakness and highlights a deficiency in the current status of research in the field.

The identification of just four articles and the initial development stages of research reported, with primary research undertaken in one geographical region, supports the need for advancement in research and practice in the development of community level health literacy assessment.

In relation to the approach used, all articles included refer to the use of a public health approach to community health literacy assessment with Guyzs *et al*. (2015) specifying the role of health promotion within this. The role of health promotion in these developments does appear apposite and is reflected in Im and Swans’s (2019) community engagement approach. The bottom-up approach working with the community taken by Im and Swan (2019) to identify domains of critical health literacy by a refugee population is in contrast to the top-down approach employed by Platter *et al*. (2019, 2022). These are not mutually exclusive approaches. Laverack (2004) presents a ‘parallel track’ planning framework that aligns stages of top-down health promotion programmes with those of bottom-up community empowerment initiatives with a process comprising programme and empowerment tracks. The top-down approach allows efficient use of already gathered data, access to established structures and networks optimizing resources. This type of development would also facilitate the inclusion of community members at all stages of the research process developing community level health literacy assessment and facilitate the incorporation of empowerment, identified in a concept analysis as a key feature of critical health literacy (Sykes *et al*. 2013). This is supported by participants in the research by Im and Swan (2019) who themselves identified empowerment as a domain in critical health literacy in a community context. This role of both individual and community empowerment, which are inextricably linked (Green *et al*. 2019), reinforces a health promotion approach to community level health literacy and its assessment.

Although a rigorous approach was undertaken through the research process, with the consistent use of a standard screening process of records with double-blind review. The large number of initial records identified has the potential to have resulted in some records being missed. A further challenge to identification and inclusion of records is caused by variations in the understandings and use of the terms ‘health literacy’ (Sørensen *et al*. 2012, Urstad *et al.* 2022) and ‘community’ (Tumiel-Berhalter and Kahn (2023) by authors in the field. Urstad *et al.* (2022) note the risk of missing important information about health literacy due to a high degree of inconsistency between health literacy definitions and instruments in current health literacy research. The authors acknowledge that such inconsistencies can impact on generalizability of findings of this scoping review.

## CONCLUSIONS

The identification of four eligible articles for inclusion in this scoping review reflects the nascent stage of development of community level health literacy and its assessment in practice. Nevertheless, some useful information has been garnered highlighting the need to focus on this area in research and practice. Findings have highlighted the potential usefulness of the Community Health Literacy Assessment Framework as a tool to measure community health literacy and further work to explore its usefulness across different geographic locations is warranted. The development of more tools that assess community level health literacy, within defined geographical communities, will facilitate, in turn, capacity building and empowerment of communities, strengthening health promotion and well-being. The importance of empowerment within communities as a key driver for the development of community health literacy is another important area that warrants further research.

## Supplementary Material

daaf123_Supplementary_Data

## Data Availability

No new data were created or analysed in this study. Data sharing is not applicable to this article.
