# Supplementary figures and images for: Approaches to assessment of community level health literacy: a scoping review
Source: Health Promot Int. 2025 Oct 22;40(5):daaf123. doi: 10.1093/heapro/daaf123 (PMC12543020; doi:10.1093/heapro/daaf123)

Appendix 1

The final search strategy as used in Medline is shown below:


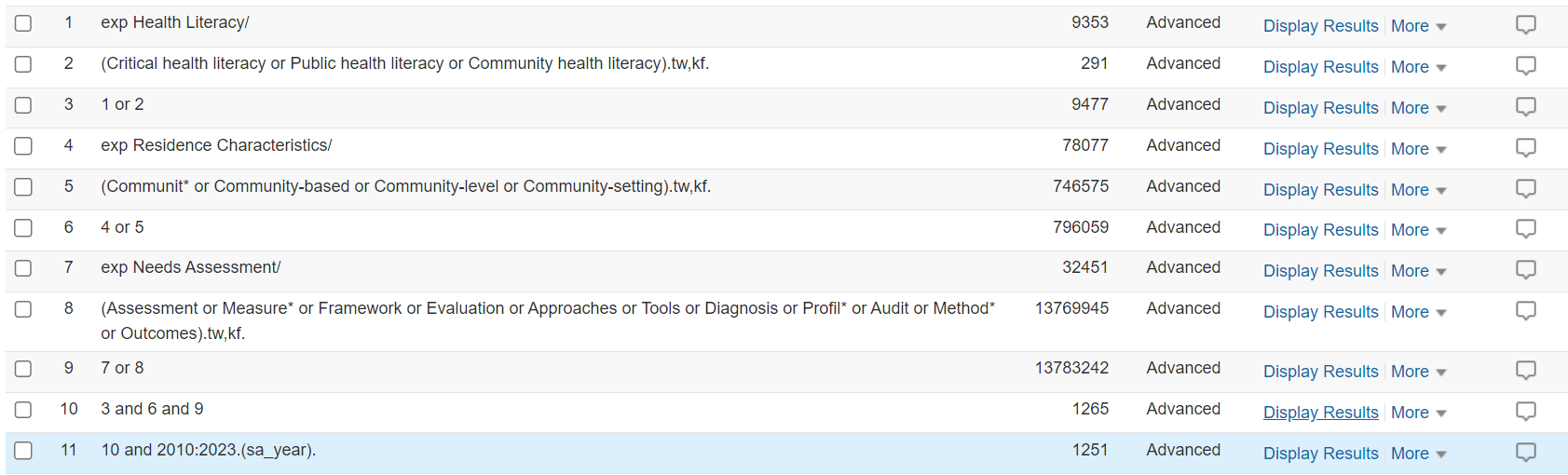

Supplement: daaf123_Supplementary_Data [file daaf123_supplementary_data.docx]
